# (3*R*,4*S*)-1-(4-Meth­oxy­phen­yl)-2-oxo-4-(3-vinyl­phen­yl)azetidin-3-yl acetate

**DOI:** 10.1107/S1600536813007897

**Published:** 2013-03-28

**Authors:** Xiao-Dong Hao, Cheng Xie, Yun-Peng Hao, Jun Chang, Xun Sun

**Affiliations:** a School of Pharmacy, Fudan University, 826 Zhangheng Road, Shanghai 201203, People’s Republic of China

## Abstract

In the title compound, C_20_H_19_NO_4_, the absolute configuration (3*R*,4*S*) for the two chiral centres of the mol­ecule has been determined.

## Related literature

For the preparation of the title compound, an inter­mediate in the synthesis of C-4 to C-3′ bridged paclitaxel analogues, see: Ganesh *et al.* (2007 ▶).
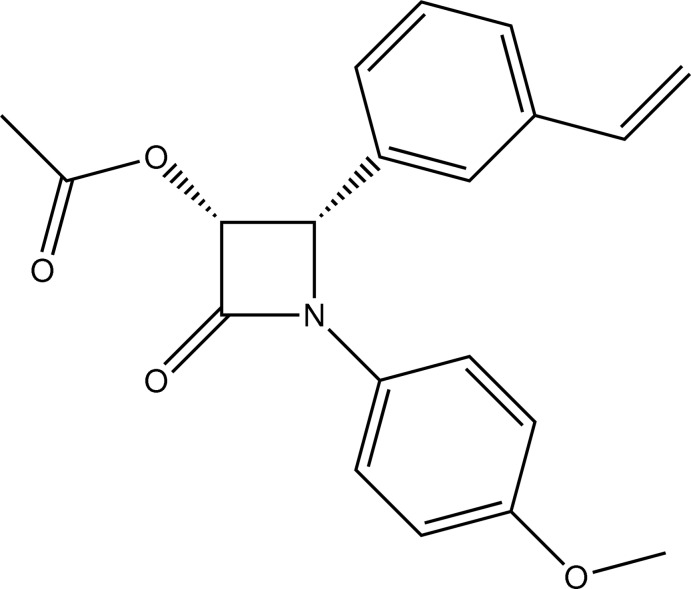


## Experimental

### 

#### Crystal data

C_20_H_19_NO_4_
*M* *_r_* = 337.36
Monoclinic, 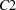

*a* = 20.7448 (4) Å
*b* = 6.3930 (1) Å
*c* = 15.7434 (3) Å
β = 124.309 (1)°
*V* = 1724.64 (5) Å^3^
*Z* = 4
Cu *K*α radiation
μ = 0.74 mm^−1^
*T* = 124 K
0.20 × 0.18 × 0.11 mm

#### Data collection

Bruker APEXII CCD diffractometer
Absorption correction: multi-scan (*SADABS*; Bruker, 2010 ▶) *T*_min_ = 0.866, *T*_max_ = 0.923
6072 measured reflections
2374 independent reflections
2330 reflections with *I* > 2σ(*I*)
*R*_int_ = 0.024

#### Refinement

*R*[*F* ^2^ > 2σ(*F* ^2^)] = 0.026
*wR*(*F* ^2^) = 0.069
*S* = 1.06
2374 reflections
228 parameters
1 restraint
H-atom parameters constrained
Δρ_max_ = 0.18 e Å^−3^
Δρ_min_ = −0.15 e Å^−3^
Absolute structure: Flack (1983 ▶), 730 Friedel pairs
Flack parameter: 0.01 (15)

### 

Data collection: *APEX2* (Bruker, 2010 ▶); cell refinement: *SAINT* (Bruker, 2010 ▶); data reduction: *SAINT*; program(s) used to solve structure: *SHELXS97* (Sheldrick, 2008 ▶); program(s) used to refine structure: *SHELXL97* (Sheldrick, 2008 ▶); molecular graphics: *SHELXTL* (Sheldrick, 2008 ▶); software used to prepare material for publication: *SHELXTL*.

## Supplementary Material

Crystal structure: contains datablock(s) I, global. DOI: 10.1107/S1600536813007897/mw2104sup1.cif

Supplementary material file. DOI: 10.1107/S1600536813007897/mw2104Isup2.cdx

Structure factors: contains datablock(s) I. DOI: 10.1107/S1600536813007897/mw2104Isup3.hkl

Supplementary material file. DOI: 10.1107/S1600536813007897/mw2104Isup4.cdx

Supplementary material file. DOI: 10.1107/S1600536813007897/mw2104Isup5.cml

Additional supplementary materials:  crystallographic information; 3D view; checkCIF report

## Figures and Tables

**Fig. 1.**
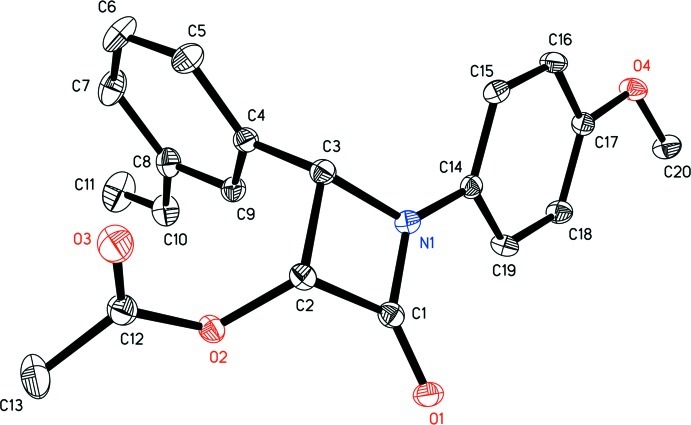
Molecular configuration and atom numbering scheme for (I).

**Fig. 2.**
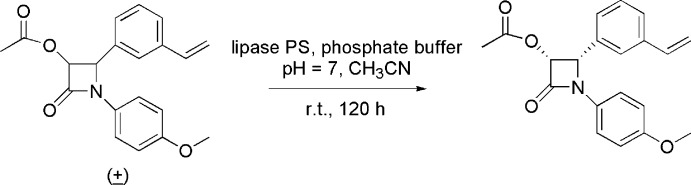
Reaction scheme for the synthesis of (I).

## supplementary crystallographic information

### Comment

In our research on the conformation of a novel fluorinated, tubulin-bound, docetaxel analogue, one of the key intermediate products, the title compound (3*R*,4*S*)-1-(4-methoxyphenyl)-2-oxo-4-(3-vinylphenyl)azetidin-3-yl acetate C_20_H_19_NO_4_ (I) (Fig. 1) was separated from the racemic 1-(4-methoxyphenyl)-2-oxo-4-(3-vinylphenyl)azetidin-3-yl acetate (Ganesh *et al.*, 2007). The reaction scheme is shown in Fig. 2. The absolute configuration (3*R*,4*S*) for the two chiral centres of the molecule has been determined.

### Experimental

Lipase PS (Amano) (2.25 g) was added to a solution of racemic 1-(4-methoxyphenyl)-2-oxo-4-(3-vinylphenyl)azetidin-3-yl acetate (2.26 g, 6.7 mmol) in 60 mL of CH_3_CN and pH 7.0 phosphate buffer (1:9), and the resulting solution was stirred at r.t. for 120 h. The reaction mixture was filtered and extracted with EtOAc (200 mL × 2), the organic layers were combined and solvent was removed under reduced pressure. The residue was purified by flash column chromatography using petroleum ether/EtOAc (4/1) to furnish the title compound (3*R*,4*S*)-1-(4-Methoxyphenyl)-2-oxo-4-(3-vinylphenyl)azetidin-3-yl acetate (1 g, 49%) as a white solid. Suitable crystals were obtained by recrystallization from hexane and DCM (m.p. 391.8–392.9 K). ^1^H NMR (400 MHz, CDCl_3_): *δ* 1.71 (s, 3H), 3.88 (s, 3H), 5.28 (d, 1H, *J* = 10.96 Hz), 5.35 (d, 1H, *J* = 4.69 Hz), 5.75 (d, 1H, *J* = 17.61 Hz), 5.95 (d, 1H, *J* = 4.70 Hz), 6.62–6.76 (m, 1H), 6.81 (d, 2H, *J* = 9 Hz), 7.18–7.42 (m, 6H); ^13^C NMR (100 MHz, CDCl_3_): *δ* 19.85, 55.45, 61.42, 76.32, 114.45, 114.80, 118.82, 125.86, 126.46, 127.28, 128.72, 130.31, 132.73, 136.20, 137.88, 156.66, 161.32, 169.24; ESIMS *m*/*z* 338.0 [*M* + H]^+^.

### Refinement

All hydrogen atoms were positioned geometrically and treated as riding with C—H = 0.95–1.00Å and *U*_iso_(H) = 1.2 or 1.5*U*_eq_(C). The title compound was identified as (3*R*,4*S*)-1-(4-methoxyphenyl)-2-oxo-4-(3-vinylphenyl)azetidin-3-yl acetate.

### Crystal data

**Table:**

C_20_H_19_NO_4_	*F*(000) = 712
*M**_r_* = 337.36	*D*_x_ = 1.299 Mg m^−^^3^
Monoclinic, *C*2	Cu *K*α radiation, λ = 1.54178 Å
Hall symbol: C 2y	Cell parameters from 4349 reflections
*a* = 20.7448 (4) Å	θ = 3.4–66.0°
*b* = 6.3930 (1) Å	µ = 0.74 mm^−^^1^
*c* = 15.7434 (3) Å	*T* = 124 K
β = 124.309 (1)°	Block, colourless
*V* = 1724.64 (5) Å^3^	0.20 × 0.18 × 0.11 mm
*Z* = 4	

### Data collection

**Table:**

Bruker APEXII CCD diffractometer	2374 independent reflections
Radiation source: fine-focus sealed tube	2330 reflections with *I* > 2σ(*I*)
Graphite monochromator	*R*_int_ = 0.024
φ and ω scans	θ_max_ = 66.3°, θ_min_ = 3.4°
Absorption correction: multi-scan (*SADABS*; Bruker, 2010)	*h* = −24→24
*T*_min_ = 0.866, *T*_max_ = 0.923	*k* = −7→6
6072 measured reflections	*l* = −18→18

### Refinement

**Table:**

Refinement on *F*^2^	Secondary atom site location: difference Fourier map
Least-squares matrix: full	Hydrogen site location: inferred from neighbouring sites
*R*[*F*^2^ > 2σ(*F*^2^)] = 0.026	H-atom parameters constrained
*wR*(*F*^2^) = 0.069	*w* = 1/[σ^2^(*F*_o_^2^) + (0.0432*P*)^2^ + 0.3527*P*] where *P* = (*F*_o_^2^ + 2*F*_c_^2^)/3
*S* = 1.06	(Δ/σ)_max_ = 0.024
2374 reflections	Δρ_max_ = 0.18 e Å^−^^3^
228 parameters	Δρ_min_ = −0.15 e Å^−^^3^
1 restraint	Absolute structure: Flack (1983), 730 Friedel pairs
Primary atom site location: structure-invariant direct methods	Flack parameter: 0.01 (15)

### Special details

**Table:**

Geometry. All e.s.d.'s (except the e.s.d. in the dihedral angle between two l.s. planes) are estimated using the full covariance matrix. The cell e.s.d.'s are taken into account individually in the estimation of e.s.d.'s in distances, angles and torsion angles; correlations between e.s.d.'s in cell parameters are only used when they are defined by crystal symmetry. An approximate (isotropic) treatment of cell e.s.d.'s is used for estimating e.s.d.'s involving l.s. planes.
Refinement. Refinement of *F*^2^ against ALL reflections. The weighted *R*-factor *wR* and goodness of fit *S* are based on *F*^2^, conventional *R*-factors *R* are based on *F*, with *F* set to zero for negative *F*^2^. The threshold expression of *F*^2^ > σ(*F*^2^) is used only for calculating *R*-factors(gt) *etc*. and is not relevant to the choice of reflections for refinement. *R*-factors based on *F*^2^ are statistically about twice as large as those based on *F*, and *R*- factors based on ALL data will be even larger.

### Fractional atomic coordinates and isotropic or equivalent isotropic displacement parameters (Å^2^)

**Table:**

	*x*	*y*	*z*	*U*_iso_*/*U*_eq_	
N1	0.01643 (6)	0.3030 (2)	0.64077 (8)	0.0200 (3)	
O1	−0.02784 (6)	0.65258 (18)	0.59931 (8)	0.0270 (2)	
O2	−0.06828 (5)	0.45389 (17)	0.74357 (7)	0.0222 (2)	
O3	−0.15201 (7)	0.2201 (2)	0.73583 (9)	0.0383 (3)	
O4	0.26072 (6)	0.13686 (18)	0.59965 (8)	0.0273 (2)	
C1	−0.02761 (7)	0.4753 (3)	0.62602 (10)	0.0211 (3)	
C2	−0.07416 (7)	0.3539 (2)	0.65865 (10)	0.0211 (3)	
H2	−0.1292	0.3251	0.6003	0.025*	
C3	−0.01748 (7)	0.1671 (2)	0.68246 (10)	0.0207 (3)	
H3	−0.0451	0.0426	0.6380	0.025*	
C4	0.03811 (7)	0.1098 (2)	0.79424 (10)	0.0211 (3)	
C5	0.02826 (8)	−0.0778 (3)	0.82961 (12)	0.0296 (3)	
H5	−0.0121	−0.1714	0.7832	0.035*	
C6	0.07760 (9)	−0.1290 (3)	0.93339 (12)	0.0390 (4)	
H6	0.0704	−0.2572	0.9576	0.047*	
C7	0.13680 (9)	0.0046 (3)	1.00140 (12)	0.0360 (4)	
H7	0.1695	−0.0314	1.0722	0.043*	
C8	0.14908 (8)	0.1925 (3)	0.96712 (11)	0.0269 (3)	
C9	0.09860 (8)	0.2431 (3)	0.86306 (11)	0.0229 (3)	
H9	0.1057	0.3711	0.8387	0.028*	
C10	0.21420 (9)	0.3354 (3)	1.03509 (12)	0.0332 (4)	
H10	0.2162	0.4619	1.0050	0.040*	
C11	0.26974 (10)	0.3070 (4)	1.13267 (14)	0.0499 (5)	
H11A	0.2706	0.1832	1.1667	0.060*	
H11B	0.3090	0.4102	1.1692	0.060*	
C12	−0.11309 (8)	0.3737 (3)	0.77324 (12)	0.0272 (3)	
C13	−0.10664 (12)	0.5031 (4)	0.85648 (14)	0.0436 (5)	
H13A	−0.1589	0.5291	0.8408	0.065*	
H13B	−0.0816	0.6367	0.8613	0.065*	
H13C	−0.0752	0.4286	0.9221	0.065*	
C14	0.07884 (7)	0.2651 (2)	0.63024 (9)	0.0191 (3)	
C15	0.09304 (7)	0.0624 (2)	0.61245 (10)	0.0224 (3)	
H15	0.0609	−0.0497	0.6071	0.027*	
C16	0.15445 (8)	0.0252 (2)	0.60258 (11)	0.0244 (3)	
H16	0.1643	−0.1127	0.5902	0.029*	
C17	0.20168 (8)	0.1904 (2)	0.61088 (10)	0.0210 (3)	
C18	0.18780 (8)	0.3919 (3)	0.62910 (11)	0.0239 (3)	
H18	0.2203	0.5038	0.6353	0.029*	
C19	0.12576 (8)	0.4292 (3)	0.63829 (10)	0.0236 (3)	
H19	0.1156	0.5673	0.6501	0.028*	
C20	0.30559 (8)	0.3045 (3)	0.59786 (13)	0.0295 (3)	
H20A	0.2707	0.4030	0.5431	0.044*	
H20B	0.3433	0.2482	0.5850	0.044*	
H20C	0.3335	0.3771	0.6642	0.044*	

### Atomic displacement parameters (Å^2^)

**Table:**

	*U*^11^	*U*^22^	*U*^33^	*U*^12^	*U*^13^	*U*^23^
N1	0.0204 (5)	0.0178 (6)	0.0214 (5)	−0.0002 (5)	0.0116 (4)	0.0004 (5)
O1	0.0313 (5)	0.0195 (6)	0.0351 (5)	0.0038 (4)	0.0218 (5)	0.0041 (5)
O2	0.0229 (4)	0.0220 (6)	0.0253 (5)	−0.0024 (4)	0.0159 (4)	−0.0037 (4)
O3	0.0451 (6)	0.0320 (7)	0.0505 (7)	−0.0147 (6)	0.0347 (6)	−0.0096 (6)
O4	0.0284 (5)	0.0215 (6)	0.0406 (5)	−0.0004 (4)	0.0247 (4)	−0.0021 (5)
C1	0.0200 (6)	0.0215 (8)	0.0202 (6)	0.0002 (5)	0.0105 (5)	−0.0017 (6)
C2	0.0210 (6)	0.0212 (8)	0.0206 (6)	0.0000 (6)	0.0116 (5)	−0.0020 (6)
C3	0.0210 (6)	0.0193 (8)	0.0238 (6)	−0.0015 (5)	0.0138 (5)	−0.0010 (6)
C4	0.0207 (6)	0.0206 (8)	0.0245 (7)	0.0014 (5)	0.0143 (5)	0.0010 (6)
C5	0.0266 (7)	0.0272 (9)	0.0322 (7)	−0.0042 (6)	0.0149 (6)	0.0028 (7)
C6	0.0380 (8)	0.0379 (11)	0.0357 (8)	−0.0049 (8)	0.0176 (7)	0.0149 (9)
C7	0.0289 (8)	0.0483 (12)	0.0268 (7)	0.0004 (7)	0.0133 (6)	0.0107 (8)
C8	0.0217 (7)	0.0350 (10)	0.0245 (7)	0.0005 (6)	0.0133 (6)	0.0001 (7)
C9	0.0230 (6)	0.0228 (8)	0.0260 (7)	0.0000 (6)	0.0156 (6)	0.0013 (6)
C10	0.0278 (7)	0.0408 (11)	0.0300 (7)	−0.0045 (7)	0.0157 (6)	−0.0031 (8)
C11	0.0360 (9)	0.0674 (15)	0.0308 (8)	−0.0154 (9)	0.0094 (7)	−0.0001 (10)
C12	0.0281 (7)	0.0256 (9)	0.0331 (7)	−0.0024 (7)	0.0204 (6)	−0.0017 (7)
C13	0.0543 (10)	0.0499 (13)	0.0450 (9)	−0.0151 (9)	0.0392 (9)	−0.0142 (9)
C14	0.0195 (6)	0.0203 (8)	0.0170 (6)	0.0018 (5)	0.0099 (5)	0.0019 (6)
C15	0.0236 (6)	0.0186 (8)	0.0251 (7)	−0.0012 (6)	0.0138 (6)	0.0021 (6)
C16	0.0281 (7)	0.0167 (8)	0.0308 (7)	0.0020 (6)	0.0180 (6)	−0.0006 (6)
C17	0.0201 (6)	0.0219 (8)	0.0215 (6)	0.0016 (5)	0.0119 (5)	0.0008 (6)
C18	0.0230 (6)	0.0198 (8)	0.0295 (7)	−0.0030 (6)	0.0153 (6)	−0.0013 (7)
C19	0.0244 (6)	0.0178 (8)	0.0282 (7)	0.0004 (6)	0.0147 (6)	−0.0037 (6)
C20	0.0267 (7)	0.0256 (8)	0.0433 (8)	0.0009 (6)	0.0240 (7)	0.0048 (7)

### Geometric parameters (Å, º)

**Table:**

N1—C1	1.365 (2)	C8—C10	1.475 (2)
N1—C14	1.4157 (17)	C9—H9	0.9500
N1—C3	1.4834 (18)	C10—C11	1.313 (2)
O1—C1	1.208 (2)	C10—H10	0.9500
O2—C12	1.3547 (18)	C11—H11A	0.9500
O2—C2	1.4237 (17)	C11—H11B	0.9500
O3—C12	1.194 (2)	C12—C13	1.491 (2)
O4—C17	1.3760 (16)	C13—H13A	0.9800
O4—C20	1.4300 (19)	C13—H13B	0.9800
C1—C2	1.535 (2)	C13—H13C	0.9800
C2—C3	1.566 (2)	C14—C19	1.387 (2)
C2—H2	1.0000	C14—C15	1.392 (2)
C3—C4	1.5077 (19)	C15—C16	1.388 (2)
C3—H3	1.0000	C15—H15	0.9500
C4—C5	1.385 (2)	C16—C17	1.396 (2)
C4—C9	1.393 (2)	C16—H16	0.9500
C5—C6	1.392 (2)	C17—C18	1.385 (2)
C5—H5	0.9500	C18—C19	1.394 (2)
C6—C7	1.379 (3)	C18—H18	0.9500
C6—H6	0.9500	C19—H19	0.9500
C7—C8	1.398 (3)	C20—H20A	0.9800
C7—H7	0.9500	C20—H20B	0.9800
C8—C9	1.397 (2)	C20—H20C	0.9800
			
C1—N1—C14	133.38 (13)	C11—C10—H10	116.4
C1—N1—C3	96.24 (11)	C8—C10—H10	116.4
C14—N1—C3	130.15 (13)	C10—C11—H11A	120.0
C12—O2—C2	116.20 (12)	C10—C11—H11B	120.0
C17—O4—C20	116.91 (12)	H11A—C11—H11B	120.0
O1—C1—N1	133.42 (13)	O3—C12—O2	123.08 (14)
O1—C1—C2	135.29 (13)	O3—C12—C13	126.19 (15)
N1—C1—C2	91.28 (12)	O2—C12—C13	110.73 (14)
O2—C2—C1	110.50 (12)	C12—C13—H13A	109.5
O2—C2—C3	117.53 (11)	C12—C13—H13B	109.5
C1—C2—C3	86.35 (10)	H13A—C13—H13B	109.5
O2—C2—H2	113.2	C12—C13—H13C	109.5
C1—C2—H2	113.2	H13A—C13—H13C	109.5
C3—C2—H2	113.2	H13B—C13—H13C	109.5
N1—C3—C4	114.66 (11)	C19—C14—C15	120.34 (12)
N1—C3—C2	85.81 (11)	C19—C14—N1	120.06 (14)
C4—C3—C2	116.23 (12)	C15—C14—N1	119.60 (13)
N1—C3—H3	112.5	C16—C15—C14	119.60 (14)
C4—C3—H3	112.5	C16—C15—H15	120.2
C2—C3—H3	112.5	C14—C15—H15	120.2
C5—C4—C9	119.27 (13)	C15—C16—C17	119.98 (14)
C5—C4—C3	119.38 (13)	C15—C16—H16	120.0
C9—C4—C3	121.34 (14)	C17—C16—H16	120.0
C4—C5—C6	119.86 (15)	O4—C17—C18	124.20 (13)
C4—C5—H5	120.1	O4—C17—C16	115.37 (13)
C6—C5—H5	120.1	C18—C17—C16	120.42 (13)
C7—C6—C5	120.61 (16)	C17—C18—C19	119.50 (14)
C7—C6—H6	119.7	C17—C18—H18	120.2
C5—C6—H6	119.7	C19—C18—H18	120.2
C6—C7—C8	120.59 (14)	C14—C19—C18	120.15 (15)
C6—C7—H7	119.7	C14—C19—H19	119.9
C8—C7—H7	119.7	C18—C19—H19	119.9
C9—C8—C7	118.18 (14)	O4—C20—H20A	109.5
C9—C8—C10	118.85 (15)	O4—C20—H20B	109.5
C7—C8—C10	122.94 (14)	H20A—C20—H20B	109.5
C4—C9—C8	121.45 (15)	O4—C20—H20C	109.5
C4—C9—H9	119.3	H20A—C20—H20C	109.5
C8—C9—H9	119.3	H20B—C20—H20C	109.5
C11—C10—C8	127.16 (19)		
			
C14—N1—C1—O1	−0.2 (3)	C6—C7—C8—C9	1.7 (2)
C3—N1—C1—O1	174.55 (16)	C6—C7—C8—C10	−176.31 (17)
C14—N1—C1—C2	−179.17 (13)	C5—C4—C9—C8	−0.6 (2)
C3—N1—C1—C2	−4.44 (11)	C3—C4—C9—C8	178.51 (13)
C12—O2—C2—C1	173.69 (12)	C7—C8—C9—C4	−0.9 (2)
C12—O2—C2—C3	−89.52 (15)	C10—C8—C9—C4	177.16 (14)
O1—C1—C2—O2	−56.7 (2)	C9—C8—C10—C11	−175.47 (18)
N1—C1—C2—O2	122.26 (11)	C7—C8—C10—C11	2.5 (3)
O1—C1—C2—C3	−174.77 (17)	C2—O2—C12—O3	4.6 (2)
N1—C1—C2—C3	4.19 (10)	C2—O2—C12—C13	−175.14 (14)
C1—N1—C3—C4	−112.63 (13)	C1—N1—C14—C19	24.9 (2)
C14—N1—C3—C4	62.36 (19)	C3—N1—C14—C19	−148.25 (14)
C1—N1—C3—C2	4.37 (11)	C1—N1—C14—C15	−155.51 (14)
C14—N1—C3—C2	179.35 (13)	C3—N1—C14—C15	31.4 (2)
O2—C2—C3—N1	−115.12 (12)	C19—C14—C15—C16	−0.1 (2)
C1—C2—C3—N1	−3.86 (10)	N1—C14—C15—C16	−179.71 (12)
O2—C2—C3—C4	0.37 (19)	C14—C15—C16—C17	0.2 (2)
C1—C2—C3—C4	111.62 (13)	C20—O4—C17—C18	−5.7 (2)
N1—C3—C4—C5	−151.25 (14)	C20—O4—C17—C16	174.20 (12)
C2—C3—C4—C5	110.90 (16)	C15—C16—C17—O4	−179.83 (12)
N1—C3—C4—C9	29.63 (19)	C15—C16—C17—C18	0.1 (2)
C2—C3—C4—C9	−68.22 (17)	O4—C17—C18—C19	179.39 (12)
C9—C4—C5—C6	1.4 (2)	C16—C17—C18—C19	−0.5 (2)
C3—C4—C5—C6	−177.79 (14)	C15—C14—C19—C18	−0.3 (2)
C4—C5—C6—C7	−0.6 (3)	N1—C14—C19—C18	179.28 (12)
C5—C6—C7—C8	−0.9 (3)	C17—C18—C19—C14	0.6 (2)
